# Detecting novel cell type in single-cell chromatin accessibility data via open-set domain adaptation

**DOI:** 10.1093/bib/bbae370

**Published:** 2024-07-29

**Authors:** Yuefan Lin, Zixiang Pan, Yuansong Zeng, Yuedong Yang, Zhiming Dai

**Affiliations:** School of Computer Science and Engineering, Sun Yat-sen University, Guangzhou, 510006, China; School of Computer Science and Engineering, Sun Yat-sen University, Guangzhou, 510006, China; School of Computer Science and Engineering, Sun Yat-sen University, Guangzhou, 510006, China; School of Computer Science and Engineering, Sun Yat-sen University, Guangzhou, 510006, China; School of Computer Science and Engineering, Sun Yat-sen University, Guangzhou, 510006, China

**Keywords:** scATAC-seq, open-set domain adaptation, cell type annotation, adversarial learning

## Abstract

Recent advances in single-cell technologies enable the rapid growth of multi-omics data. Cell type annotation is one common task in analyzing single-cell data. It is a challenge that some cell types in the testing set are not present in the training set (i.e. unknown cell types). Most scATAC-seq cell type annotation methods generally assign each cell in the testing set to one known type in the training set but neglect unknown cell types. Here, we present OVAAnno, an automatic cell types annotation method which utilizes open-set domain adaptation to detect unknown cell types in scATAC-seq data. Comprehensive experiments show that OVAAnno successfully identifies known and unknown cell types. Further experiments demonstrate that OVAAnno also performs well on scRNA-seq data. Our codes are available online at https://github.com/lisaber/OVAAnno/tree/master.

## Introduction

Recent single cell sequencing technologies have provided the opportunities to facilitate the analysis of cells in complex tissues at high resolution. To analyze these data, one important task is cell types annotation [1, 2]. The general step of cell annotation is preprocessing data, removing batch effects, clustering cells through unsupervised clustering, finding gene markers, and finally assigning a cell type to each cluster based on markers. However, as the amount of data grows rapidly, so do the time and labor cost of this annotation procedure [3]. The set of markers may be different by using different methods, leading to inconsistance in the annotation results [4]. These problems motivate the development of automatic cell type annotation methods which annotate target data using annotated (i.e. labeled) source data.

Most of existing automatic cell type annotation methods focus on scRNA-seq(single-cell RNA) data, such as TOSICA [4], scmap [5], and scibet [6]. Some methods applied to scRNA-seq data can be directly applied to scATAC-seq (single-cell Assay for Transposase Accessible Chromatin) data, such as LIGER [7], scMC [8], and Seurat v3 [9]. However, the overall performance of direct application is not good, especially for methods that require converting the peak-to-cell matrix into gene-to-cell matrix (i.e. gene activity matrix), which may result in the loss of some biological information [10]. This suggests that an automatic cell type annotation model tailored to scATAC-seq data is necessary.

There are some cell type annotation methods specially designed for scATAC-seq data. Signac [11] is an end-to-end tool that enables complete analysis of scATAC-seq data, including cell type annotation and integration. Cellcano [12] firstly trains an Multilayer Perceptron (MLP) which can assign the pesudo label to the test data, and then trains a self-knowledge distiller model [13] on test data with high confidence. However, the above methods cannot predict unknown cell types; that is the cell types that exist in test data but not in training data. To the best of our konwledge, EpiAnno is the only method that can predict unknown cell types, which combines probabilistic generative model and Bayesian neural network [14]. EpiAnno constructs a multivariate Gaussian distribution for each cell type, from which embedding is sampled. Then, the embedding is reconstructed to peak-to-cell matrix. This whole process is much like Variational AutoEncoder (VAE) [15]. The weights of the model follow the set prior distribution, and in the phase of inference, the model uses its inverse function for cell type prediction by setting a fixed threshold to distinguish between known and unknown cell types. However, this strategy of fixed threshold may become problematic when batch effects vary among different batches.

There are some challenges for cell type annotation in scATAC-seq data: (i) Batch effects: differences in data distribution between source and target datasets (i.e. batches) due to technical influences during the experiment [16, 17]. (ii) The captured accessibility area can vary significantly between batches. (iii) Compared with scRNA-seq data, scATAC-seq data have higher sparsity and dimensionality, with nearly binarized values [18]. (iv) The number of cell types may vary between different batches.

Domain adaptation is a technique to improve the performance of a model on a target domain containing insufficient annotated data by using the knowledge learned by the model from a different but related source domain with adequate labeled data. The domains can be different batches in the context of single-cell data. Recently, some methods based on domain adaptation have been proposed to solve batch effects in single-cell data [19]. Zhou *et al.* [20] used pseudolabeling to minimize the class center between the training and test data for cell type annotation. Jialu *et al.*combined variational auto-encoder and adversarial learning strategy for batch correction and dimension reduction [21]. Koff *et al.* integrated scATAC-seq data in a similar way [22]. Yingxin *et al.* added variations related to confounding factors to represent batch effect and reduced its impact on the latent representation related to intrinsic cell states [23]. However, methods above generally assume that the training dataset and the test dataset have the same cell type, which can not be applied to the test set with novel cell type.

Open-set domain adaptation assumes that test set contains not only all the types in the training set but also unknown type that does not exist in training set [24]. Busto *et al.* assumed that there is also unknown type in the training set and used it to identify unknown type in the test set [24]. Through adversarial training strategy, Saito *et al.* proposed OSBP, which draws a boundary between training set and test set and treats it as the threshold between known and unknown types [25]. On the basis of OSBP, Liu *et al.* added multiple binary classifiers to progressively separate known and unknown samples [26]. Zhang *et al.* assigned weights to each sample in adversarial learning and ignored samples with smaller weights in domain alignment [27]. Sifan *et al.* selected samples with high confidence and aligned known samples by weighted adversarial learning [28]. These open-set domain adaptation methods are proposed for computer vision, while there are no similar methods for cell type annotations.

Therefore, we propose One-vs-All Annotation(OVAAnno), a deep learning model which can annotate cell type and detect novel cell types with open-set domain adaptation. The embedding with biological information extracted by our method can be applied in downstream analysis, such as discovery of cell type-specific motifs and inference of transcription factor regulatory networks.

## Methodology

**Figure 1:**
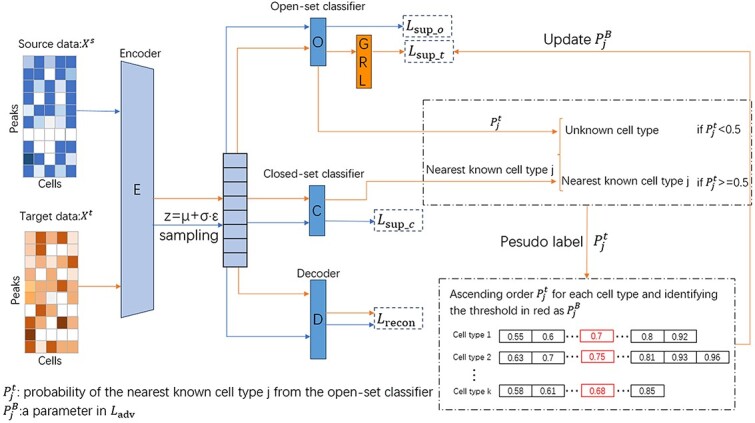
Basic framework of OVAAnno; the orange line represents the data flow of the target set and the blue line represents the data flow of the source set; the source and target data share all network parameters.

OVAAnno consists of three parts: VAE, a closed-set classifier and an open-set classifier including multiple binary classifiers (Fig. 1). Through the VAE, we can obtain an information-rich embedding. Mapping the embedding to known cell types, the closed-set classifier selects the nearest known cell type for one sample (i.e. one cell). With adversarial training strategy, the open-set classifier identifies whether the sample belongs to known or unknown cell types.

### Data preprocessing

Following the EpiAnno, for the scATAC datasets fed to model, when the peaks of the training set and those of the test set do not coincide, we map reads of training set to peaks of test set [14]. Then, we binarize the peak matrices and only reserve the peaks that have at least one read count in at least 0.1% of cells. Finally, we select 20 000 highly variable peaks as features. The value in processed peak matrix indicates whether chromatin region is open or not(value 1 for open and otherwise). For the scRNA dataset, after filtering the genes that express in less than 0.1% of cells, we normalize the dataset by normalization and log function in scanpy with a size factor of 10 000. Finally, we select 3000 highly variable genes as features.

### VAE and closed-set classifier

Here, the processed peak matrix $X\in \mathbb{R}^{N\cdot m}$ has $N$ cells and $m$ peaks (source: $X^{s}=\{x^{s}_{i}\}^{N^{s}}_{i=1}\in \mathbb{R}^{N^{s}\cdot m}$, target: $X^{t}=\{x^{t}_{i}\}^{N^{t}}_{i=1}\in \mathbb{R}^{N^{t}\cdot m}$), and each cell in source data has corresponding cell types $Y^{s}=\{y^{s}_{i}\}^{N^{s}}_{i=1}$, which includes K cell types $T_{K}=\{C_{i}\}^{K}_{i=1}$. Following the Kingma and Welling [15], we encode the data to the L-dimensional mean $\mu $ and variance $\sigma $ parameters by encoder and take the sample $Z \in \mathbb{R}^{N\cdot L}$ from the approximate posterior distribution. Then, it is reconstructed as $\hat{X} \in \mathbb{R}^{N\cdot m}$ by decoder. With sigmoid function, the output of decoder can be considered as the probability whether the region is open or not. Using the KL divergence(Equation 1), we approximate the distribution of Z to the Gaussian distributions, while using multivariable Bernoulli distribution to model the data by binary cross entropy loss($L_{recon}$). For the scRNA data, we use mean square error loss as reconstruction loss. The source and target data share the same encoder and decoder. This is an effective strategy for solving domain adaptation problem [29]. Because VAE is a generative model, the sample $Z$ can be considered as the embedding of a new sample, which can be considered as data augmentation. Meanwhile, $Z^{s}$ from the source data are fed into the closed-set classifier with the optimization goal of cross entropy loss($L_{sup{\_ }c}$).


(1) \begin{align*}& \begin{aligned} KL_{div}&= KL(\mathcal N(\mu,\sigma^{2}) || \mathcal N(0,I))\\ &=\frac{1}{2}\left(-1 - log\sigma^{2} + \mu^{2} + \sigma^{2}\right). \end{aligned}\end{align*}

### Open-set classifier on source data

Open-set classifier consists of K binary classifiers. For binary classifier j, it determines whether the sample is $C_{j}$ or not, and samples not belong to $C_{j}$ are negative samples. For each classifier, the number of negative samples may be larger than the number of positive samples. As negative samples include multiple cell types, the number of samples for each cell type in negative samples may vary greatly [30]. To solve class-imbalance problem, motivated by [31], we define the open-set classifier loss on source data as


(2) \begin{align*} L_{sup{\_}o}& = {\frac{1}{N^{s}}}\sum^{K}_{j = 1} \sum^{N^{s}}_{i = 1} -\alpha_{ij}\hat{p_{ij}}log(1 - \hat{p_{ij}}) \end{align*}

(3) \begin{align*} \hat{p_{ij}}&= \begin{cases} 1 - p(y = C_{j} | x^{s}_{i})\quad&{ if \ y_{i} = C_{j}} \\ p(y = C_{j} | x^{s}_{i})\quad&{if \ y_{i} \neq C_{j}} \end{cases} \end{align*}

(4) \begin{align*} \alpha_{ij}&= \begin{cases} \frac{\sum_{l=0}^{N} |y_{l} \neq C_{j}|}{\sum_{l=0}^{N} |y_{l} = C_{j}|} \quad&{if \ y_{i} = C_{j}}\\ 1 \quad&{if \ y_{i} \neq C_{j}}. \end{cases} \end{align*}

Here, $y_{i}$ denotes the true cell type of $x_{i}^{s}$. In Equation 2, the higher $\hat{p_{ij}}$, the higher the weight of the sample. High $\hat{p_{ij}}$ only occurs if positive samples has low probabilities or negative samples has high probabilities. That means each binary classifier pays more attention to hard-to-distinguish samples and ignores easy-to-distinguish samples. In this way, the boundary lies between the known cell type and its nearest unknown cell types, which can minimize the risk of misaccepting unknown cell types as its near known cell types. To further alleviate the bias of open-set classifier, we also use $\alpha _{ij}$ to reweight the positive sample loss by the ratio of negative and positive sample sizes.

### Open-set classifier on target data

In a previous study [25], to minimize batch effects between source data and target data, OSBP’s classifier sets a probability value boundary, which we denote it as $P_{b}$. With adversarial training strategy, the training goal of OSBP’s classifier is to make the probability value approximate $P_{b}$, while the training goal of the generator is the opposite. The generator of OSBP decreases the probability for a known class but increases the probability for an unknown class. Finally, the differences of distribution between source known class and target known class are eliminated and unknown class is pushed away from known class. However, OSBP sets $P_{b}$ as a fixed value 0.5 which may not be suitable for each datasets for the reason that the difference in distribution between different datasets may be different. More importantly, the probability of unknown class that close to known class may fall near 0.5 and be greater than 0.5. As training proceeds, the probability of the unknown class may become higher and higher, eventually with unknown class identified as known class.

Our idea is to set a customized value boundary for each binary classifier separately and the vector $P^{B}$ consisting of all value boundary is adjusted automatically with the training process. Unlike OSBP, we add Gradient Reversal layer [32] after open-set classifier to reverse the gradient, so that the probabilities of target samples are pushed away from $P^{B}$. Since the training of each binary classifier is similar, here, we use the $j$th classifier $cls_{j}$ which identifies whether target samples are cell type $C_{j}$ or not, as an example for the purpose of illustration.

As shown on the bottom right side of Fig. 1, we obtain the nearest known cell types $C_{j}$ of one target sample $i$ by closed-set classifier, and this sample will be assigned to $C_{j}$ if the corresponding probability $P_{ji}^{t}$ of $C_{j}$ from the open-set classifier is greater than 0.5, otherwise this sample will be assigned to unknown cell types. Through this process, we can obtain pseudo label $\hat{Y^{t}}$ (i.e. one specific cell type) for each target sample. The vector $P^{t}_{j}$, each element of which is the probability of each target sample assigned to $C_{j}$, is sorted in ascending order and the number at the top 5% interval of $P^{t}_{j}$ is considered as $P^{B}_{j}$. With the removal of the batch effects, both $P^{t}_{j}$ and $P^{B}_{j}$ increase in training process. For one sample $i$ not belong to $C_{j}$, its $P_{ji}^{t}$ may be higher than $P^{B}_{j}$, but generally lower than those of samples belong to $C_{j}$. As $P^{B}_{j}$ continues to rise, samples not belong to $C_{j}$ are gradually excluded from $C_{j}$. For each mini batch, after training, $\hat{Y^{t}}$ and $P^{B}$ in that mini batch are updated.


(5) \begin{align*} & \begin{aligned} L_{sup{\_}t} = {\frac{1}{N \cdot K}\sum^{K}_{j}\sum^{N}_{i}\beta_{ij} * \left(-P^{B}_{j}logp(y = C_{j}|x^{t}_{i})\right.} \\[-1em] {\left.- (1-P^{B}_{j})log(1-p(y = C_{j}|x^{t}_{i}))\right)} \end{aligned} \end{align*}

(6) \begin{align*} & \beta_{ij}= \begin{cases} 1 - p(y = C_{j}|x^{t}_{i}) \quad&{ if \ p(y = C_{j}|x^{t}_{i})> P^{B}_{j}}\\ 1 - P^{B}_{j} \quad&{ if \ 1 - P^{B}_{j} < p(y = C_{j}|x^{t}_{i}) <= P^{B}_{j}}\\ p(y = C_{j}|x^{t}_{i})\quad&{ if \ p(y = C_{j}|x^{t}_{i}) <= 1 - P^{B}_{j}}. \end{cases} \end{align*}

We also define weight $\beta $ on target data which can help model to pay more attention to the target samples whose probability values are near the $P^{B}_{j}$, ignoring a large number of samples irrelevant to $C_{j}$. Because class-imbalance may exist in the target data, the model may predict target samples belong to known cell types as unknown cell types without our reweighting strategy. Because $P^{B}_{j}$ may become higher and higher with training, the weights of samples assigned unknown cell types may increase. We set the upper limit of weight for sample whose probability is below $P^{B}_{j}$ and above $1 - P^{B}_{j}$. The training algorigthm is shown in Algorithm 1.


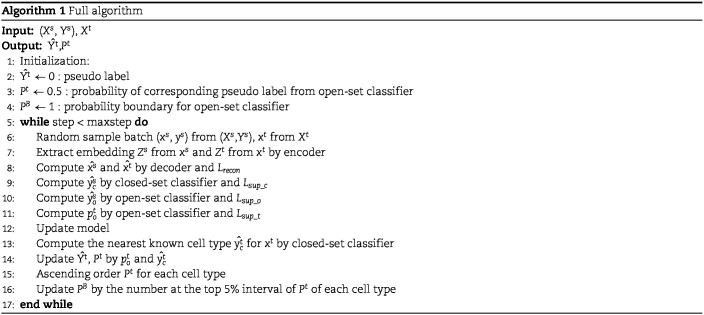


### Supervised cell type annotation methods

OVAAnno is benchmarked with three scATAC-seq cell type annotation methods, including EpiAnno, Signac [11], and Cellcano [12]. As for the scRNA-seq, we benchmark OVAAnno with ACTINN [41], Scibet [6], Scmap [5], CellBlast [42], and scvi [43]. We chose these scRNA-seq cell type annotation methods as they show outstanding performance in a previous benchmark study [44]. For these methods, we utilized their default hyperparameters. The preprocessing steps, such as selection of feature numbers, were uniformly executed utilizing the source code of each respective method unless stated otherwise. Following the benchmark method of Cellcano, we use scATAC-seq gene score matrix instead of the scATAC-seq peak matrix for all scATAC-seq methods except EpiAnno and Signac [12]. The training process of these methods is consistent with that described in original paper. For the methods than cannot directly predict unknown cell types, including scvi and Signac, we set the thresholds as the values on which the methods can perform best on all tested datasets, 0.9 for scvi and signac. For all methods except Cellcano, cells with output confidence under the threshold are assigned as unknown cell types. For Cellcano which outputs entropy at the second phase, we sort the cells descendingly by entropy and assign the top $K$ cells to unknown cell types, where $K$ is the number of unknown cells in the test dataset.

## Experiment

We collect four scATAC datasets (Table 1) for experiment. Since a dataset may contain multiple batches, we use each batch as a training or test set. Since the cell types in the Cusanovic dataset contain cell types from the other three datasets, we use Cusanovic dataset as test set and the other datasets as training set. To examine the robustness of our method, we use Cusanovic as training set and Forebrain as test set. We first remove cell types in Cusanovic but not in Forebrain, and then randomly remove one cell type in Cusanovic. In this way, the number of cell types in Forebrain is higher than that in Cusanovic. For 10X mouse brain dataset, Cusanovich dataset and Fang dataset, Luecken *et al.* used FASTQ files to reprocess them and unify their peak [10], which can be used directly for our experiment. The training configs are shown in Tables S1 and S2.

**Table 1:** Details of scATAC-seq dataset

Dataset	Number of Batchs	Range of Number of Cells	Range of Number of Cell Types	protocol	Source	train/test
10X_mouse_brain	1(10X)	3667	5	10X genomics	https://support.10xgenomics.com/single-cell-atac /datasets/1.2.0/atac_v1_adult_brain_fresh_5k	train
Fang	6(FA,FB,FC,FD, FE,FF)	9967-11034	6	snATAC-seq	http://data.nemoarchive.org/biccn/grant/cemba/ecker/chromatin/scell/raw/	train
Forebrain [33]	1(Fore)	1098	5	snATAC-seq	GSM2668124	train
Cusanovich [34]	4(Cel,Pre,WholeA, WholeB)	2278-5959	20-22	sci-ATAC-seq	https://atlas.gs.washington.edu/mouse-atac/data/	test

**Table 2:** Details of scRNA-seq dataset

Dataset	Number of Batchs	Range of Number of Cells	Range of Number of Cell Types	protocol	Source
Melanoma [35]	1	6173	8	SMART-Seq2	https://singlecell.broadinstitute.org/single_cell/study/SCP109/melanoma-immunotherapy-resistance
Macaque [36]	2(Peri,Fovea)	9285-21017	12	10X Genomics	GSE118546
Baron_Human [37]	1(Baron)	8569	14	inDrop	GSE84133
Xin [38]	1	1492	4	SMARTer	GSE81608
Segerstolpe [39]	1	2068	9	Smart-seq2	E-MTAB-5061
Muraro [40]	1	2042	8	CEL-Seq2	GSE85241

### Implementation and evaluation metric

For scATAC-seq experiments, encoder is a neural network including four layers (3200-1600-800-400). The embedding size is 32. Decoder, closed-set classifier, and open-set classifier are directly connected to embedding without hidden layer. Mini batch size is 64 and training epochs is 4000. Learning rate is 0.0002 with AdamW optimizer, weight_decay is 5e$-4$. The difference of scRNA-seq experiments is that the encoder is three-layer neural network (256-128-128) and weight_decay is 5e$-5$.

Two evaluation metrics, effective assignment score (EAS) and effective multiple assignment score (EAS_M), are used in our experiments. We define $N_{k}$ as the number of test samples that belong to known cell types, $N_{u}$ as the number of test samples that belong to unknown cell types, $N_{k}^{k}$ as the number of samples in $N_{k}$ predicted to be any of the known cell types, and $N_{u}^{k}$ as the number of samples in $N_{u}$ predicted to be any of the known cell types. In a previous study [14], EAS is defined as Equation 7. However, EAS reflects only whether a sample is assigned to known cell types or not, but not whether a sample is assigned to the corresponding correct cell type or not. We thus propose EAS_M(Equation 8). In EAS_M, $N_{k}^{true}$ represent the number of samples in $N_{k}$ predicted to be their true cell types. For example, training set has two cell types A and B. Test set has three cell types A, B, and C. In EAS, it is correct that cells of cell type A are predicted as cell type A or B. But in EAS_M, the prediction is correct only when cells of cell type A are predicted as cell type A. EAS_M is a stricter metric than EAS.


(7) \begin{align*} & \begin{aligned} EAS = \frac{N_{k}^{k}}{N_{k}} - \frac{N_{u}^{k}}{N_{u}} \end{aligned} \end{align*}

(8) \begin{align*} & \begin{aligned} EAS{\_}M = \frac{N_{k}^{true}}{N_{k}} - \frac{N_{u}^{k}}{N_{u}}. \end{aligned} \end{align*}

We used Cohen’s kappa, which is a minor-class sensitive approach thus can give us a comprehensive evaluation of classification performance, including the major types identification and the minor types identification. This metric is based on the confusion matrix, and its equation is


(9) \begin{align*}& \begin{aligned} Kappa &= \frac{p_{o} - p_{e}}{1 - p_{e}}\\ p_{o} &= \frac{\sum_{i = 1}^{N}A_{ii}}{\sum_{i = 1}^{N}\sum_{j = 1}^{N}A_{ij}}\\ p_{e} &= \frac{\sum_{i = 1}^{N}\left(\left(\sum_{j = 1}^{N}A_{ij}\right) * \left(\sum_{j = 1}^{N}A_{ji}\right)\right)}{\left(\sum_{i = 1}^{N}\sum_{j = 1}^{N}A_{ij}\right)^{2}}, \end{aligned}\end{align*}

where $A_{ij}$ represents the element in the $i$th row and $j$th column of the confusion matrix A, with the total number of samples being $N$.

### Classification performance

As shown in Table 3, OVAAnno outperforms all benchmark methods on most datasets. Although OVAAnno and other methods get a lower score in EAS_M than EAS, OVAAnno shows more similar performance between EAS_M and EAS compared with EpiAnno. This shows that OVAAnno not only distinguishes known cell types from unknown cell types more accurately but also accurately predicts samples as corresponding known cell types.

**Table 3:** Performance comparison of methods on scATAC-seq data

Dataset	EAS				EAS_M				Kappa			
	EpiAnno	Signac	Cellcano	OVAAnno	EpiAnno	Signac	Cellcano	OVAAnno	EpiAnno	Signac	Cellcano	OVAAnno
ForeToCel	0.62	0.0045	0.3360	**0.6974**	0.4799	−0.0437	0.0745	**0.6801**	0.579	0.2006	0.3015	**0.7433**
ForeToPre	0.45	0.4695	0.4292	**0.4709**	0.4369	**0.4581**	0.2189	0.4563	0.7285	0.7443	0.5911	**0.7713**
ForeToWholeA	0.59	0.0957	0.4138	**0.6586**	0.5481	0.0722	0.2893	**0.648**	0.69	0.3795	0.4804	**0.7334**
ForeToWholeB	0.64	0.2146	0.4068	**0.7278**	0.5654	0.2002	0.3091	**0.7164**	0.7057	0.4596	0.502	**0.7931**
10XToCel	−0.0501	0.186	0.0785	**0.5617**	−0.1309	0.1647	−0.1499	**0.5119**	0.332	0.3798	0.1519	**0.5939**
10XToPre	0.1369	**0.8253**	−0.0009	0.5092	0.0735	**0.8032**	−0.1557	0.4786	**0.8454**	0.7016	0.7544	0.5073
10XToWholeA	−0.0406	0.4159	0.4109	**0.6132**	−0.1325	0.3628	0.2856	**0.5773**	0.4476	0.5462	0.3593	**0.6298**
10XToWholeB	−0.0643	0.4776	0.3612	**0.5726**	−0.1563	0.4527	0.2389	**0.5309**	0.4772	**0.6352**	0.3842	0.5877
FAToCel	0	0.1008	0.1529	**0.5411**	−0.2408	0.0902	0.0067	**0.4758**	0.2535	0.4113	0.2056	**0.5365**
FAToPre	0.1389	**0.7315**	−0.0009	0.4476	−0.0311	**0.7056**	−0.0975	0.3661	0.6793	0.5944	**0.8372**	0.576
FAToWholeA	−0.0296	0	0.3756	**0.6751**	−0.3221	−0.0213	0.2714	**0.549**	0.2934	0.4139	0.3695	**0.6449**
FAToWholeB	−0.048	−0.0031	0.353	**0.5679**	−0.3955	−0.0216	0.2592	**0.4306**	0.291	0.4693	0.4051	**0.6113**
FBToCel	−0.0321	0.0369	0.1287	**0.5332**	−0.1219	0.0246	0.0013	**0.4409**	0.3227	0.3895	0.2096	**0.6193**
FBToPre	0.1089	**0.8529**	0.1658	0.1117	−0.1476	**0.8148**	0.032	−0.0931	0.5499	0.5893	**0.7777**	0.3315
FBToWholeA	−0.0484	−0.0262	0.3463	**0.7692**	−0.1796	−0.0617	0.2418	**0.606**	0.3752	0.4318	0.3624	**0.6911**
FBToWholeB	−0.0926	−0.0339	0.3295	**0.7168**	−0.2244	−0.0633	0.2384	**0.546**	0.3946	0.4657	0.4015	**0.674**
FCToCel	0	−0.001	0.0751	**0.6339**	−0.1567	0.0882	−0.0652	**0.509**	0.3249	0.3805	0.1894	**0.6615**
FCToPre	−0.0001	**0.7016**	−0.0009	0.5016	−0.2143	**0.6752**	−0.1276	0.3919	0.6742	0.5623	**0.7899**	0.6022
FCToWholeA	0	−0.0619	0.3561	**0.7249**	−0.1954	−0.0763	0.2374	**0.6036**	0.4077	0.3684	0.3561	**0.6892**
FCToWholeB	0	−0.0210	0.3377	**0.6477**	−0.2741	−0.0350	0.2317	**0.5289**	0.4	0.4327	0.3916	**0.6727**
FDToCel	0	0.0889	0.0059	**0.6549**	−0.1265	0.0791	−0.1499	**0.4859**	0.3418	0.3946	0.1666	**0.6307**
FDToPre	−0.029	**0.8133**	−0.0009	0.2382	−0.1651	**0.7814**	−0.1025	0.1486	0.7423	0.6761	**0.829**	0.5159
FDToWholeA	−0.021	−0.0379	0.314	**0.545**	−0.1237	−0.0565	0.1954	**0.3933**	0.4515	0.4018	0.3438	**0.5206**
FDToWholeB	−0.0276	−0.0164	0.3571	**0.5565**	−0.1386	−0.0345	0.2674	**0.4128**	0.4929	0.4525	0.3809	**0.5641**
FEToCel	−0.0244	0.1141	0.1062	**0.6031**	−0.1542	0.1027	−0.0227	**0.4431**	0.322	0.4103	0.2022	**0.6124**
FEToPre	0.1384	**0.7741**	0.1658	0.4291	0.0426	**0.752**	0.074	0.351	0.7968	0.6455	**0.8458**	0.5601
FEToWholeA	−0.0328	−0.0157	0.3741	**0.6479**	−0.1616	−0.0309	0.2725	**0.5474**	0.4269	0.4186	0.3703	**0.6373**
FEToWholeB	−0.0606	−0.0369	0.3571	**0.6576**	−0.1798	−0.0519	0.2674	**0.5407**	0.4597	0.4586	0.4082	**0.6498**
FFToCel	−0.0059	0.0739	0.1892	**0.6287**	−0.0884	0.0584	0.0537	**0.4621**	0.3579	0.4037	0.2172	**0.6015**
FFToPre	−0.0137	**0.8678**	0.1658	0.2422	−0.1311	**0.8241**	0.0453	0.1754	0.7911	0.57	**0.8008**	0.5439
FFToWholeA	−0.0091	−0.0098	0.4041	**0.5705**	−0.1091	−0.0485	0.3066	**0.4743**	0.4613	0.402	0.3812	**0.5624**
FFToWholeB	−0.0291	−0.0105	0.4207	**0.5845**	−0.1293	−0.0463	0.3278	**0.4825**	0.4963	0.4532	0.4223	**0.5985**
CelToFore	0	−0.046	−0.0357	**0.2388**	−0.8014	−0.3251	−0.4799	**0.2388**	0.0781	−0.0159	0.1637	**0.2527**
PreToFore	−0.0462	0.0304	**0.3888**	0.2837	−0.0673	−0.3071	**0.3802**	0.2775	0.3622	0.0162	**0.6133**	0.3191
WholeAToFore	0.3845	0.0423	0.5475	**0.597**	0.3337	−0.2282	0.5326	**0.5945**	0.5870	0.0229	**0.7198**	0.5593
WholeBToFore	0.0044	0.0161	0.4681	**0.5421**	−0.2114	−0.2221	0.4458	**0.5421**	0.4228	0.0241	**0.6725**	0.4872

Dataset name annotation: #To*(# is training set, * is test set)

When we use 10X or six batchs in Fang dataset as training sets, respectively(Table 3), OVAAnno still performs better than other methods in most datasets. EpiAnno’s performance drops dramatically. This may be due to the large batch effects between training set and test set. We also combined each batch from Fang and 10X as the training set (Table S3). The results show that even though the training data with batch effects generated by different sequencing technologies, OVAAnno still performs better than other methods in general. Note that the number of cells of unknown cell types of Pre batch is only six, and identifying these rare unknown cell types is a challenged task for most methods. Signac performs well on Pre. This is because, at very high thresholds, Signac filters out almost all unknown types, resulting in relatively few cells of known types being predicted as unknown. Although Signac can perform best on this task with our chosen optimal threshold, it lacks robustness on other datasets and it is impractical to manually choose optimal threshold for every dataset.

As shown in Figs S1a and S2a, some cells of the same cell type are not clustered together because of batch effect. Compared with Cellcano (Fig. S1c) and Signac (Fig. S1e), OVAAnno can better separate unknown cell type with other cell types (Fig. S1b). EpiAnno appears to perform well in both integration and clustering. However, compared with ground truth (Fig. S1a), EpiAnno predicts too many unknown types as known types (Fig. S1d), which may be due to negative transfer. An interesting observation for Signac output is that cells belong to excitatory neurons cell type are not clustered together (Fig. S1e), and a possible explanation is that multiple subtypes of excitatory neurons exist in the cortex.

On most datasets, OVAAnno performs better on the Kappa metric compared with other methods. Meanwhile, Cellcano significantly outperforms other methods on Pre, suggesting that it shows promise in predicting rare cell types.

### Ablation experiment

In this experiment, using Fore batch, FA batch as training sets, respectively, and Cel batch, Pre batch as test sets, respectively, we evaluate the influence of $L_{sup{\_ }o}$ and $P^{B}$ on OVAAnno(Table 4). To evaluate the influence of $L_{sup{\_ }o}$, we replace it with traditional binary cross entropy for each binary classifier in open-set classifier. Without $L_{sup{\_ }o}$, it is hard for OVAAnno to draw a clear boundary between known cell types and unknown cell types for each binary classifier. As more samples of unknown cell types may have probability values higher than $P^{B}$, OVAAnno w/o $L_{sup{\_ }o}$ tends to identify samples of unknown cell types as known cell types. From Table 4, OVAAnno’s performance declines when $L_{sup{\_ }o}$ is replaced.

**Table 4:** Ablation experiments

method	ForeToCel		FAToCel		FAToPre	
	EAS	EAS_M	EAS	EAS_M	EAS	EAS_M
OVAAnno w/o $L_{sup{\_ }o}$	0.6936	0.6626	0.4306	0.3457	0.1431	0.0399
OVAAnno with fixed $P^{B}$	0.0707	–0.008	–0.1165	0.116	0.2028	0.1076
OVAAnno w/o $\beta $	0.0037	0.0037	0.0439	0.0431	0.0335	0.0322
OVAAnno w/o $L_{sup{\_ }t}$	0.3239	0.2384	0.2854	0.1074	0.1349	0.1106
OVAAnno	**0.6974**	**0.6801**	**0.5411**	**0.4758**	**0.4476**	**0.3661**

To demonstrate the effectiveness of adjusted $P^{B}$ during training, we replace it with fixed value 0.5. Without adjusted $P^{B}$, the performance of OVAAnno drops drastically, as samples of unknown cell types are more likely to be misassigned to known cell types. It is possible for OVAAnno to filter the unknown cell types that close to known cell types by adjusted $P^{B}$.

To demonstrate the effectiveness of weight $\beta $, we remove it during training. As shown in Table 4, OVAAnno’s performance drops dramatically. For open-set binary classifier which identifies whether target samples are cell type $C_{j}$ or not, other cell types are considered as unknown cell types. There are far more cells of unknown cell types than $C_{j}$. So the binary classifier tends to predict that the sample belongs to unknown cell types without $\beta $.

To demonstrate the effectiveness of loss $L_{sup{\_ }t}$, we remove it during training. From Table 4, the performance of model drops significantly without $L_{sup{\_ }t}$. without $L_{sup{\_ }t}$, open-set classifier can only identify the cell types in the source data. For example, if source data has cell type $C_{a}$ and $C_{b}$, but not includes cell type $C_{c}$ which exists in target data. For a binary classifier which identify whether a cell is $C_{a}$, the binary classifier can identify $C_{b}$ as unknown cell type through supervised learning but it may not be able to distinguish $C_{c}$ for the reason that source data have little information about $C_{c}$. $L_{sup{\_ }t}$ can make the classifier effectively separate all cells that do not belong to $C_{a}$, including the cell types not existing in source data.

### Effect of parameter selection

**Figure 2:**
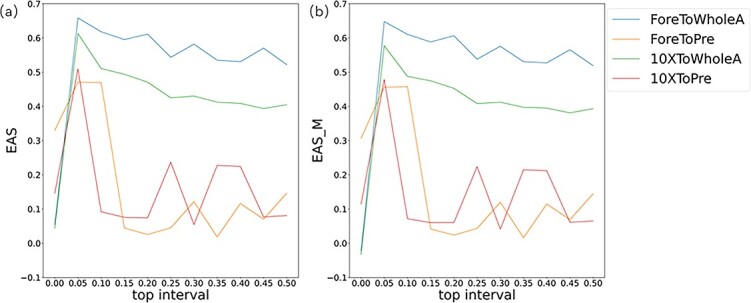
Effect of parameter selection; (a and b) the choice of top interval for Pb on model performance.

We use four training sets to test the impact of changes in top interval selection for $P^{B}$ on the model. As shown in Fig. 2a and b, OVAAnno performs best when we select the probability value at the top 5% boundary as $P^{B}$. When top interval is set as 0, unknown cell types similar to cell type $C_{j}$ may be assigned pesudo label $C_{j}$ in open-set classifier, which makes the performance of OVAAnno poor.

We also examined the effects of different numbers of highly variable peaks on model performance (Tables S4 and S5). When we select 20 000 highly variable peaks for each cell, OVAAnno performs best on most datasets. The 10X dataset has 3667 cells, 81 of which is Endothelial Cell. When the 10X dataset is used as training data (10XToWholeA and 10XToPre), the performance of OVAAnno does not apparently increase with increasing number (>20 000) of peaks. In the default preprocessing steps of other methods, they all retained far more than 20 000 features. We varied the number of features for these methods. Cellcano, however, utilized all peak information, converting the peak matrix into a gene activity matrix. Due to the smaller number of genes, this experiment could not be performed for Cellcano. It can be observed that an increase in the number of features does not necessarily enhance the performance of these methods. On the contrary, it may introduce noise, thereby degrading performance.

**Table 5:** Performance comparison of methods on scRNA-seq data

method	MTrainToMTest			PeriToFovea			XSMToBaron			BaronToXSM			Average		
	EAS	EAS_M	Kappa	EAS	EAS_M	Kappa	EAS	EAS_M	Kappa	EAS	EAS_M	Kappa	EAS	EAS_M	Kappa
Scibet	0.6586	0.6096	0.6774	0.5452	0.5357	0.6378	0.1206	−0.8753	0.1017	0.3474	−0.2458	0.6792	0.418	0.0061	0.524
Scmap-cluster	−0.2038	−0.2527	0.1376	0	0	0	0.3763	0.3567	0.3474	0.8369	0.813	0.7929	0.2523	0.2293	0.3194
Scmap-cell	−0.1594	−0.2439	0.0884	0.4114	0.3913	0.3897	0.4666	0.4302	0.8371	0.5137	0.4526	0.8310	0.3081	0.2576	0.5365
ACTINN	0.7622	0.7504	0.7918	**0.6508**	**0.6503**	0.6140	0.6526	0.6332	0.6101	0.8001	0.7548	0.7087	0.7164	0.6972	0.6811
scvi	0.4395	0.3762	0.4849	0.4423	0.4375	**0.9824**	0.5974	0.5674	**0.9028**	0.0103	−0.0312	0.8705	0.3723	0.3374	0.8101
CellBlast	0.7551	0.6867	0.7148	0.2719	0.2482	0.6690	0.5855	0.5653	0.5907	0.0200	−0.0436	**0.8706**	0.4081	0.3641	0.7112
OVAAnno	**0.9196**	**0.863**	**0.8818**	0.5411	0.5273	0.9304	**0.7201**	**0.6917**	0.7982	**0.8946**	**0.8609**	0.8454	**0.7688**	**0.7357**	**0.8365**

Dataset name annotation: #To*(# is training set, * is test set)

### ScRNA-seq experiment

We further examined the performance of our model on scRNA data by collecting six datasets (Table 2). For melanoma dataset, following the setting of a previous study [6], 70% of immune cells are randomly sampled as training set and the others as test set, denoted as MTrain and MTest, respectively. Peri batch of Macaque dataset is selected as training set and Fovea is selected as test set. Xin, Segerstolpe, and Muraro are combined into a new dataset XSM. Then following a previous study [20], XSM cells labeled as unclear, co-expression, unclassified, unclassified endocrine, alpha.contaminated, beta.contaminated, delta.contaminated, or gamma.contaminated are removed. When using XSM as training set and Baron as test set, those cell types available in XSM but not in Baron are removed, and a similar operation is performed when using XSM as the test set and Baron as the training set. For MTrainToMTest and PeriToFovea datasets, as the training and test sets are from the same source, there are small batch effects. On the other hand, for XSMToBaron and BaronToXSM datasets, the training and test sets are from different sources. The training configs are shown in Tables S6–S9.

From Table 5, OVAAnno outperform other methods in most datasets. In the four datasets tested, the performance of OVAAnno is the top on three datasets and is the third on the other dataset which has small batch effects between training and test sets. For XSMToBaron and BaronToXSM, Baron and XSM are produced by different scRNA-seq sequence technologies, leading to large batch effects between training and test sets. Moreover, XSM consists of three different datasets produced by different scRNA-seq technologies, so there are batch effects in XSM. OVAAnno can accurately identify unknown cell types in these experiments.

## Disscussion and conclusion

In summary, we proposed OVAAnno, an automatic cell type annotation method combined with open-set domain adaptation for scATAC-seq data. In this work, not only simply removing batch effects, our method can also isolate unknown samples. Moreover, it can be applied to scRNA-seq data, which suggests that it can be used on single-cell data of different modalities.

Our future work is to extend the method so that it can be applied to multimodal data, such as scATAC data and scRNA-seq data. One limitation is that our method shows moderate performance on datasets with small batch effects, the case that one dataset is split into training and test sets. Extending the application of our method to various types of datasets is a direction of improvement.

**Key Points**

- OVAAnno sets an automatically adjusted threshold to separate known samples and unknown samples.
- OVAAnno sets a closed-set classifier which identifies the specific type of known sample to help open-set classifier to identify unknown samples.
- OVAAnno is an end-to-end method for automatic cell type annotation based on open-set domain adaptation.

## Supplementary Material

Supplementary_material_bbae370
