# Supplementary material for: Detecting novel cell type in single-cell chromatin accessibility data via open-set domain adaptation
Source: Brief Bioinform. 2024 Jul 29;25(5):bbae370. doi: 10.1093/bib/bbae370 (PMC11285170; doi:10.1093/bib/bbae370)
Supplement: Supplementary_material_bbae370 [file supplementary_material_bbae370.pdf]

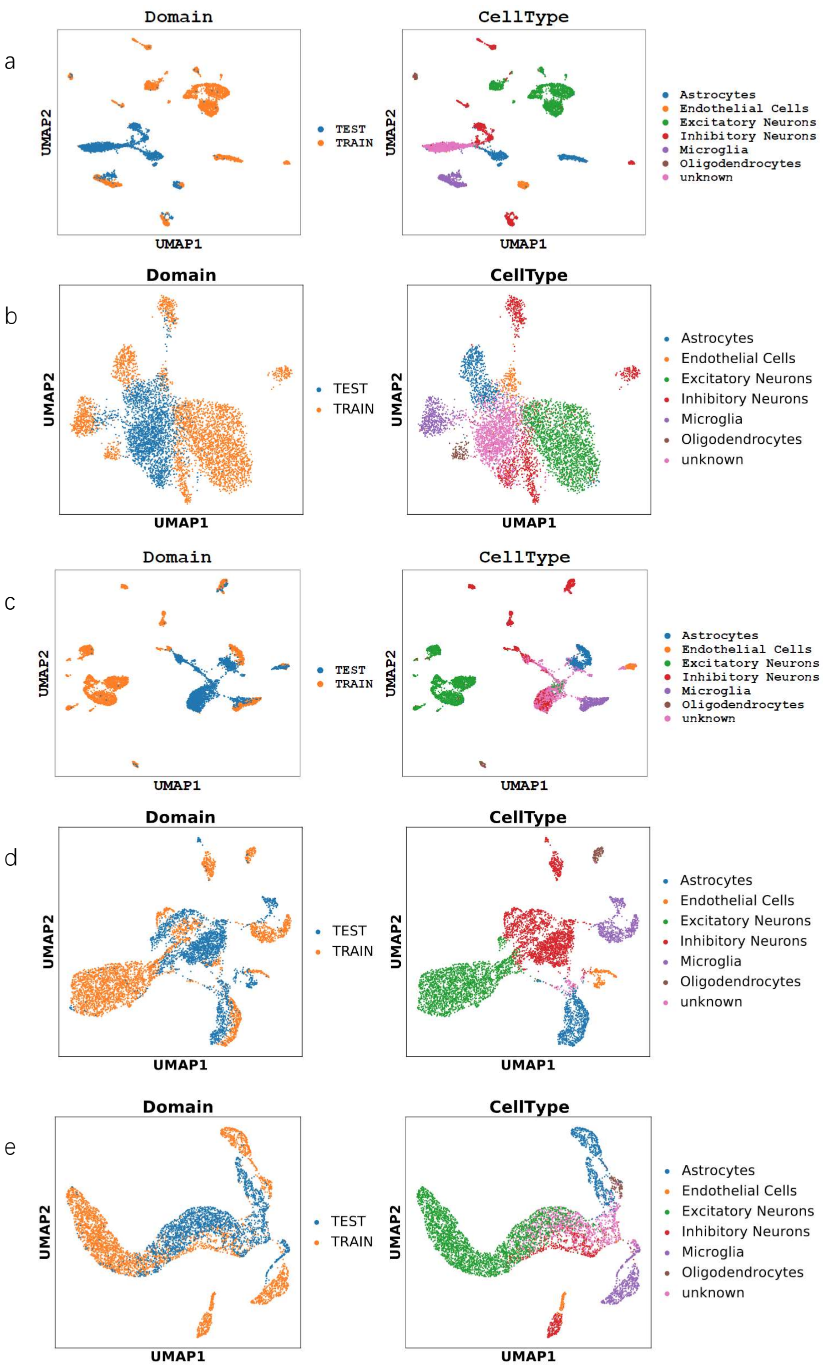

Fig. S1. Qualitative evaluation of four methods using UMAP visualization for 10XToCel dataset. The four methods are organized into five rows, showing UMAP plots of (a) raw data, (b) OVAAnno, (c) Signac, (d) EpiAnno, (e) CellCano outputs. Each row contains two UMAP plots. In the left column, cells are colored by batch, and in the right column by cell type.

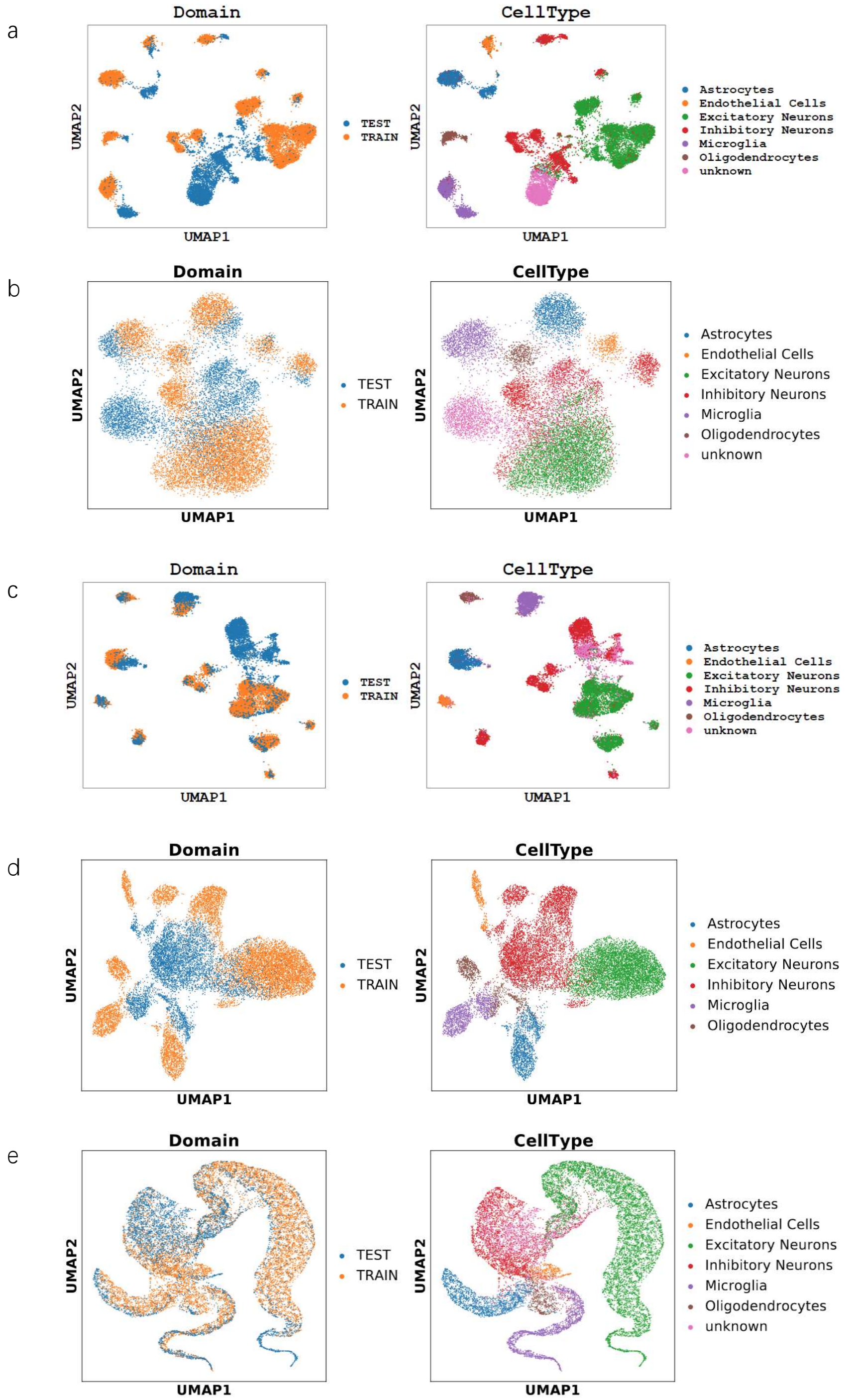

Fig. S2. Qualitative evaluation of four methods using UMAP visualization for FCToWholeA dataset. The four methods are organized into five rows, showing UMAP plots of (a) raw data, (b) OVAAnno, (c) Signac, (d) EpiAnno, note that EpiAnno predicts no cells that belong to unknown cell type. (e) CellCano outputs. Each row contains two UMAP plots. In the left column, cells are colored by batch, and in the right column by cell type.

Table S1. Training config of Forebrain and Cusanovich

Dataset	train/test	Batch	CellType					
			AC	EX	IN	MG	OC	unknown
Forebrain	train	Fore	120	1075	515	126	252	0
Cusanovich	test	Cel	363	6	281	29	128	1471
		Pre	551	3546	320	197	458	887
		WholeA	401	806	839	110	575	2763
		WholeB	348	479	366	81	394	1604

Table S2. Training config of 10X mouse brain, Fang and Cusanovich

Dataset	train/test	Batch	CellType						
			Astrocytes	Oligodendrocytes	Endothelial Cells	Excitatory Neurons	Inhibitory Neurons	Microglia	Cerebellar Granule Cells
10X mouse brain	train	10X	357	102	81	2038	669	420	0
Fang	train	FA	1565	636	1069	5028	2259	477	0
		FB	1534	522	1053	4629	1868	361	0
		FC	1142	518	327	5158	2229	920	0
		FD	1020	509	293	5556	2532	1071	0
		FE	1209	537	288	5078	2523	1254	0
		FF	1301	652	393	4589	2172	1433	0
Cusanovich	test	Cel	445	5	112	14	499	150	1096
		Pre	627	145	127	3520	1123	475	6
		WholeA	508	107	129	857	1454	696	2063
		WholeB	423	45	112	533	612	482	1076

Table S3. Performance comparison of methods on batch-combined training data

	EAS				EAS_M				Kappa			
	EpiAnno	Signac	cellcano	OVAAnno	EpiAnno	Signac	cellcano	OVAAnno	EpiAnno	Signac	cellcano	OVAAnno
10x_FAToCel	-0.1036	-0.1009	0	**0.5632**	-0.4987	-0.1123	-0.4082	**0.4628**	0.0773	0.2558	0.2698	**0.6183**
10x_FAToPre	0.6091	**0.8267**	0	0.3569	0.306	**0.7967**	-0.11	0.2967	0.2616	0.6967	**0.8218**	0.6847
10x_FAToWholeA	-0.2566	-0.0219	0	**0.6812**	-0.4491	-0.0582	-0.1706	**0.6044**	0.1529	0.3903	0.4272	**0.6762**
10x_FAToWholeB	0	-0.0583	0	**0.6089**	-0.4884	-0.0905	-0.1744	**0.5409**	0.2328	0.4355	0.4687	**0.6693**
10x_FBToCel	0	-0.0915	0	**0.6562**	0	-0.1053	-0.3812	**0.5207**	0	0.2636	0.2821	**0.6543**
10x_FBToPre	0.5847	**0.6336**	0	0.1877	0.3449	**0.6121**	-0.117	0.1155	0.2964	0.6763	**0.8105**	0.6667
10x_FBToWholeA	0.5217	0.0412	0	**0.7101**	0.2695	0.0137	-0.168	**0.6285**	0.3863	0.415	0.4306	**0.7024**
10x_FBToWholeB	-0.0009	0.0387	0	**0.6366**	-0.5043	0.0133	-0.179	**0.5663**	0.2125	0.4699	0.4659	**0.6734**
10x_FCToCel	0	-0.0567	0	**0.6265**	-0.4604	-0.0738	-0.3951	**0.4796**	0.14	0.2629	0.2707	**0.6321**
10x_FCToPre	0.493	**0.6622**	0	0.3913	0.269	**0.6306**	-0.108	0.3094	0.4026	0.6967	**0.8242**	0.6961
10x_FCToWholeA	0	0.1439	0	**0.6941**	-0.4385	0.1045	-0.1818	**0.6104**	0.2194	0.4571	0.4209	**0.6936**
10x_FCToWholeB	0	0.016	0	**0.6955**	-0.5002	-0.0135	-0.1636	**0.6071**	0.226	0.447	0.4775	**0.7109**
10x_FDToCel	0	-0.0579	0	**0.6336**	-0.4841	-0.0726	-0.4171	**0.4858**	0.1307	0.2807	0.262	**0.6272**
10x_FDToPre	0	**0.6568**	0	-0.0002	-0.5195	**0.6284**	-0.1193	-0.0737	0.2967	0.6953	**0.8071**	0.6366
10x_FDToWholeA	0.0107	-0.063	0	**0.6204**	-0.4033	-0.0966	-0.1874	**0.5426**	0.2368	0.3639	0.4158	**0.638**
10x_FDToWholeB	0.262	-0.0413	0	**0.5985**	0.2253	-0.0735	-0.1903	**0.493**	0.2892	0.4359	0.4558	**0.6286**
10x_FEToCel	-0.0122	-0.0519	0	**0.6574**	-0.3829	-0.0682	-0.3347	**0.5276**	0.1813	0.2836	0.3044	**0.6577**
10x_FEToPre	0.2928	**0.6518**	0	0.1911	0.1638	**0.6269**	-0.1143	0.1259	0.4737	0.6935	**0.8149**	0.6761
10x_FEToWholeA	0.5997	0.1123	0	**0.6724**	0.2496	0.0781	-0.1602	**0.5879**	0.4633	0.4439	0.4368	**0.6832**
10x_FEToWholeB	0.5396	-0.0817	0	**0.5937**	0.2555	-0.1044	-0.1563	**0.4999**	0.4013	0.4274	0.4831	**0.6505**
10x_FFToCel	0	0.0087	0	**0.598**	-0.4971	-0.0068	-0.3763	**0.4551**	0.1298	0.2952	0.2826	**0.5968**
10x_FFToPre	0	**0.812**	0	0.341	-0.5617	**0.7861**	-0.1205	0.2735	0.2646	0.6859	**0.8065**	0.645
10x_FFToWholeA	0	0.0764	0	**0.6402**	-0.4623	0.0457	-0.1578	**0.5632**	0.2175	0.4188	0.4363	0.6494
10x_FFToWholeB	-0.0009	0.0816	0	**0.6197**	-0.4957	0.0548	-0.1649	**0.54**	0.2372	0.4726	0.4753	0.6418

Dataset name annotation: #1_#2To*(#1 is training set 1, #2 is training set 2, * is test set)

Table S4. The EAS of different feature values on various methods.

Dataset	10XToCel			10XToPre			ForeToPre			ForeToWholeA		
Peak_num	OVAAnno	EpiAnno	Signac	OVAAnno	EpiAnno	Signac	OVAAnno	EpiAnno	Signac	OVAAnno	EpiAnno	Signac
5000	0.0814	0.0117	0.0056	-0.0329	0.0901	0.0553	0.3215	0.2268	0.3278	0.4128	0.2601	0.3323
10000	-0.0348	**0.3517**	0.0138	-0.0851	0.0011	0.2017	0.5283	0.3611	**0.6678**	0.5928	0.4654	0.5042
15000	0.4707	0.0078	0.0653	0.3422	**0.1938**	0.3147	0.4157	0.5157	0.6286	0.648	0.6205	0.5522
20000	**0.5617**	-0.011	0.1427	0.5092	-0.0363	0.4269	**0.4709**	**0.5157**	0.5932	**0.6586**	**0.6205**	**0.567**
25000	0.4663	-0.0038	0.2187	0.4977	0.1186	0.5255	0.4238	0.5157	0.512	0.6567	0.6205	0.511
30000	0.4035	-0.0096	0.3172	0.3316	0.1134	0.5858	0.4231	0.5157	0.4201	0.6515	0.6205	0.5528
35000	0.331	0.0019	0.3581	0.3366	0.135	0.6268	0.4695	0.5157	0.398	0.6548	0.6205	0.3835
40000	0.3588	0.0213	**0.4029**	**0.522**	0.1443	**0.6774**	0.4082	0.5157	0.3821	-0.0477	0.6205	0.295

Table S5. The EAS_M of different feature values on various methods.

Dataset	10XToCel			10XToPre			ForeToPre			ForeToWholeA		
Peak_num	OVAAnno	EpiAnno	Signac	OVAAnno	EpiAnno	Signac	OVAAnno	EpiAnno	Signac	OVAAnno	EpiAnno	Signac
5000	-0.3552	-0.1563	-0.0009	-0.4848	-0.0643	0.0462	0.2407	0.0099	0.3183	0.2872	-0.002	0.2419
10000	-0.2846	**0.1272**	0.0073	-0.2385	-0.1833	0.1947	0.5056	0.315	**0.6611**	0.5726	0.1791	0.4691
15000	0.3466	-0.1643	0.0653	0.2972	**0.028**	0.3082	0.4005	0.5039	0.619	0.6352	0.5462	0.5431
20000	**0.5119**	-0.1506	0.1411	0.4786	-0.2089	0.4164	0.4563	**0.5039**	0.582	0.648	0.5462	**0.5597**
25000	0.441	-0.1516	0.2171	0.475	-0.0409	0.5143	0.41	0.5039	0.5025	**0.649**	**0.5462**	0.503
30000	0.3961	-0.1402	0.3156	0.3163	-0.0404	0.5722	0.4072	0.5039	0.409	0.6445	0.5462	0.5448
35000	0.3155	-0.1106	0.3557	0.3218	-0.0133	0.6127	**0.4569**	0.5039	0.3861	0.6485	0.5462	0.3729
40000	0.34	-0.0806	**0.3955**	**0.507**	-0.0008	**0.6609**	0.3907	0.5039	0.371	-0.0631	0.5462	0.2818

Table S6. Training config of Melanoma

Dataset	train/test	Batch	CellType					
			B.cell	Macrophage	NK	T.CD4	T.CD8	unknown
Melanoma	train	MTrain	573	294	64	599	1231	0
Melanoma	test	MTest	245	126	28	257	528	2228

Table S7. Training config of Macaque

Dataset	train/test	Batch	CellType											
			BB/GB*	DB1	DB2	DB3a	DB3b	DB4	DB5*	DB6	FMB	IMB	RB	unknown
Macaque	train	Peri	468	376	613	294	540	732	919	216	797	1007	3323	0
Macaque	test	Fovea	1347	620	1631	329	2100	2253	2548	442	3703	5144	753	147

Table S8. Training config of Baron and XSM

Dataset	train/test	Batch	CellType									
			acinar	alpha	beta	delta	ductal	endothelial	epsilon	gamma	mast	unknown
Baron	train	Baron	958	2326	2525	601	1077	252	18	255	25	0
XSM	test	XSM	404	2584	1190	356	631	37	10	383	7	139

Table S9. Training config of Baron and XSM(2)

Dataset	train/test	Batch	CellType									
			acinar	alpha	beta	delta	ductal	endothelial	epsilon	gamma	mast	unknown
XSM	train	XSM	404	2584	1190	356	631	37	10	383	7	0
Baron	test	Baron	958	2326	2525	601	1077	252	18	255	25	532
